# Financial Hardship, Hope, and Life Satisfaction Among Un/Underemployed Individuals With Psychiatric Diagnoses: A Mediation Analysis

**DOI:** 10.3389/fpsyt.2022.867421

**Published:** 2022-07-22

**Authors:** Oscar Jiménez-Solomon, Ryan Primrose, Ingyu Moon, Melanie Wall, Hanga Galfalvy, Pablo Méndez-Bustos, Amanda G. Cruz, Margaret Swarbrick, Taína Laing, Laurie Vite, Maura Kelley, Elizabeth Jennings, Roberto Lewis-Fernández

**Affiliations:** ^1^New York State Psychiatric Institute, Columbia University Irving Medical Center, New York, NY, United States; ^2^Center on Poverty and Social Policy, School of Social Work, Columbia University, New York, NY, United States; ^3^Teacher’s College, Columbia University, New York, NY, United States; ^4^Nyack College, New York, NY, United States; ^5^Department of Biostatistics, Mailman School of Public Health, Columbia University Irving Medical Center, New York, NY, United States; ^6^Department of Psychiatry, Vagelos College of Physicians and Surgeons, Columbia University, New York, NY, United States; ^7^Department of Psychology, Faculty of Health Sciences, Catholic University of Maule, Talca, Chile; ^8^Department of Psychology, Clark University, Worcester, MA, United States; ^9^Center of Alcohol and Substance Use Studies, Graduate School of Applied and Professional Psychology, Rutgers University – The State University of New Jersy, Piscataway, NJ, United States; ^10^Collaborative Support Programs of New Jersey, Freehold, NJ, United States; ^11^Baltic Street AEH, Inc., Brooklyn, NY, United States; ^12^Mental Health Peer Connection, Western New York Independent Living, Buffalo, NY, United States; ^13^National Disability Institute, Washington, DC, United States

**Keywords:** financial hardship, hope, life satisfaction, unemployment, poverty

## Abstract

**Background:**

Individuals with psychiatric diagnoses who are unemployed or underemployed are likely to disproportionately experience financial hardship and, in turn, lower life satisfaction (LS). Understanding the mechanisms though which financial hardship affects LS is essential to inform effective economic empowerment interventions for this population.

**Aim:**

To examine if subjective financial hardship (SFH) mediates the relationship between objective financial hardship (OFH) and LS, and whether hope, and its agency and pathways components, further mediate the effect of SFH on LS among individuals with psychiatric diagnoses seeking employment.

**Methods:**

We conducted structured interviews with participants (*N* = 215) of two peer-run employment programs using indicators of OFH and SFH and standardized scales for hope (overall hope, hope agency, and hope pathways) and LS. Three structural equation models were employed to test measurement models for OFH and SFH, and mediational relationships. Covariates included gender, age, psychiatric diagnosis, race/ethnicity, education, income, employment status, SSI/SSDI receipt, and site.

**Results:**

Confirmatory factor analysis (CFA) for items measuring OFH and SFH supported two separate hypothesized factors. OFH had a strong and significant total effect on SFH [standardized beta (*B*) = 0.68] and LS (*B* = 0.49), and a weak-to-moderate effect on hope (*B* = –0.31). SFH alone mediated up to 94% of the effect of OFH on LS (indirect effect *B* = –0.46, *p* < 0.01). The effect of SFH on LS through hope was small (indirect effect *B* = –0.09, *p* < 0.05), primarily through hope agency (indirect effect *B* = –0.13, *p* < 0.01) and not hope pathways. Black and Hispanic ethno-racial identification seemed to buffer the effect of financial hardship on hope and LS. Individuals identifying as Black reported significantly higher overall hope (*B* = 0.41–0.47) and higher LS (*B* = 0.29–0.46), net of the effect of OFH and SFH.

**Conclusion:**

SFH is a strong mediator of the relationship between OFH and LS in our study of unemployed and underemployed individuals with psychiatric diagnoses. Hope, and particularly its agency component, further mediate a modest but significant proportion of the association between SFH and LS. Economic empowerment interventions for this population should address objective and subjective financial stressors, foster a sense of agency, and consider the diverse effects of financial hardship across ethno-racial groups.

## Introduction

*Financial hardship*, usually defined as difficulty meeting basic needs and financial responsibilities, has emerged as an important construct in mental health (1–4). People with psychiatric diagnoses are more likely than the general population to undergo financial hardship, as they experience a higher prevalence of poverty, unemployment, underemployment, and dependency on public benefits with income and asset poverty requirements (1, 3–7). Consequently, people with psychiatric diagnoses experience substantial difficulty meeting basic needs (1) and paying utility bills (8, 9), face considerable food and housing insecurity (10, 11), and often endure over-indebtedness (8, 9, 12).

The high prevalence of financial hardship among people with psychiatric diagnoses is particularly important because financial hardship can adversely impact *subjective wellbeing, life satisfaction (LS)*, and mental health (13–15). Subjective wellbeing has been defined as the extent to which people *believe and feel* that their lives are going well (16) and is based on cognitive evaluations (e.g., LS) and affects (e.g., joy, happiness) (17). LS refers to people’s explicit and conscious evaluations of their lives, which are based on life domains that matter to the individual (16). Across low- and high-income countries, people living in poverty experience significantly lower subjective wellbeing (18, 19). While low income significantly predicts subjective wellbeing (18), financial hardship may be an even stronger predictor (20, 21). Longitudinally, financial hardship predicts worsening mental health over time (4); moreover, financial hardship and psychological distress seem to have a reciprocal relationship that creates a cycle of socio-economic decline and mental health deterioration (15, 22, 23). In fact, financial hardship may be one of the most important social determinants of mental health and the strongest single socio-economic predictor of poor mental health (2).

Research has highlighted the association of specific hardship indicators – inability to make ends meet, difficulty paying bills on time, housing insecurity, and over-indebtedness – and poor subjective wellbeing (20, 24–28). For instance, difficulty meeting basic needs has a negative impact on subjective wellbeing (21, 29) and LS in particular (30); difficulty paying monthly bills has also been associated with lower LS (31). Housing hardship (e.g., difficulty paying for housing, high housing cost–income ratio) is associated with lower subjective wellbeing (32, 33) and strongly predicts lower satisfaction with life (34). Research has also found a robust association between indebtedness and LS (24, 25, 35, 36), which is moderated by type and level of debt (24). Credit card debt, for instance, is associated with lower subjective wellbeing more consistently than housing or education debt (9, 37). Similarly, higher debt amount and higher debt-to-income ratios, which are likely to cause debt unmanageability, heighten the negative effect of indebtedness (9, 38).

*Unemployment* and *underemployment* have a particularly negative impact on subjective wellbeing. Cross-sectional and longitudinal meta-analyses in the past twenty years have found that unemployment has a moderate-to-strong effect on psychological distress and overall psychological wellbeing, and that unemployed individuals are on average over twice as likely to report psychological problems than employed individuals (39, 40). Longer periods of unemployment worsen its effect on mental health (40, 41). People with psychiatric diagnoses may be particularly vulnerable to the effects of unemployment and underemployment. Employment status is a strong predictor of LS in this population (42, 43). Hence, the negative effect of psychiatric diagnoses on LS (44) is compounded by the financial and non-financial effects of unemployment (45–47). Accounting for socio-demographic factors and the presence of depression, financial hardship has a moderate negative effect on the LS of unemployed individuals (48). This effect of unemployment on LS is only partly related to its effect on income loss (45, 46). Employment fulfills not only material but also psychological needs, such as structure, social connections, and life purpose (49). As a result, unemployment can be experienced as a loss of control, agency, and social status, with important psychological and social consequences beyond its economic impact (50). Nevertheless, the relationship between unemployment and wellbeing may be bidirectional, since lower self-rated health increases the chance of joining the pool of the unemployed (51).

Underemployment also has detrimental effects on LS and mental health. Although evidence about the effect of underemployment on wellbeing is limited (52), longitudinal research has shown that transitioning from full-time employment to underemployment (defined as 30 h of work or less per week) predicts increases in psychological distress, while individuals who move out of underemployment experience reductions in distress (53). When underemployment is defined by wage changes, underemployed individuals report LS and mental health improvements relative to those who are unemployed. However, when underemployment is defined based on utilization of skills, the underemployed show no better LS than the unemployed. While income improvements of paid employment may have benefits, being overqualified for a job may create life dissatisfaction and distress (54).

Despite the consistent association between financial hardship indicators and LS, the mechanisms of this association among unemployed and underemployed individuals with psychiatric diagnoses are insufficiently understood. Research in the general population suggests that *subjective financial hardship (SFH)* – one’s own experience of a difficult financial situation – may be an important mechanism of the impact of objective financial hardship (OFH) on LS. SFH has been variously defined in the literature. Defined as *financial satisfaction* (a person’s overall evaluation of how pleased they are with their financial situation), it is a moderate-to-strong predictor of LS (55, 56). As *financial stress* (anxiety and worry about one’s financial situation) and *financial threat* (uncertainty and vulnerability associated with financial difficulties), it is a moderate predictor of poor LS (57, 58). Furthermore, SFH partially mediates the effect of objective indicators on subjective wellbeing. For example, *perceived financial wellbeing* mediates the effect of low cash reserves on LS (59). *Financial dissatisfaction* mediates the impact of indebtedness on low satisfaction with life (35). In a recent large meta-analysis, *subjective socioeconomic status* (SES) partly mediates the association of objective SES and subjective wellbeing (13). The *perception of one’s financial situation* (dissatisfaction, perceived ability to control one’s financial situation, perceived financial future) mediates the relationship between OFH and psychological wellbeing (60). *Shame* about one’s financial circumstances, a common experience in response to financial hardship and poverty (61, 62), mediates the relationship between financial hardship and anxiety (63). Among the unemployed, shame and financial hardship seem to interact to intensify the detrimental effect of unemployment on mental wellbeing (64). Furthermore, shame can be not only a consequence of financial hardship but also exacerbate it. Shame can induce *financial withdrawal* (disengagement from financial actions to improve one’s financial situation) and thereby prevent individuals from addressing their financial difficulties (65).

Another important construct in this nexus of associations related to financial hardship and LS is a sense of *hope*, which may also mediate their association (66, 67). Snyder’s Hope Theory defines hope as a positive motivational state resulting from two interrelated sets of cognitions: *pathways* and *agency*. *Pathways* refers to the perceived capacity to generate workable routes to achieve one’s valued goals, while *agency* is motivational as it relates to the perceived capacity to initiate action along such pathways and even switch pathways when a barrier is experienced (68). Hopeful thinking occurs when both components are assessed and re-assessed continuously by the individual, in interaction with their environment, as they pursue their personal goals (69). A recent longitudinal study found that hope mediates the association between income and LS among moderate-income individuals and between income and happiness among high-income individuals. Hope agency was a stronger predictor than hope pathways (67). In another study, hope mediated the relationship between SFH and depression, stress, and wellbeing (63). These findings are consistent with research showing that feelings of hopelessness partially mediate the relationship between debt and suicidal ideation (70); they are also consistent more generally with research on the link between hope and subjective wellbeing, especially the moderate-to-strong positive association found between hope and LS (71–73).

Taken together, the literature suggests that indicators of OFH – such as difficulty meeting basic needs and paying bills on time, housing unaffordability, and over-indebtedness – may be important predictors of LS among unemployed and underemployed individuals with psychiatric diagnoses (24, 31, 34). Experiencing OFH can foster dissatisfaction, worry, uncertainty, and shame about one’s financial situation (14, 35, 55). In turn, the subjective experience of financial hardship may erode individuals’ hope about their ability to achieve personal goals, leading to poorer satisfaction with life (63). For some, such as individuals already struggling with their mental health and those disengaged from the workforce, this process may lead to worsened psychiatric distress and deteriorating mental health. Nevertheless, our understanding about these mechanisms among people with psychiatric diagnoses is still incipient and the available evidence insufficient to develop effective interventions to buffer the impact of financial hardship on the subjective wellbeing of this population.

**FIGURE 1:**
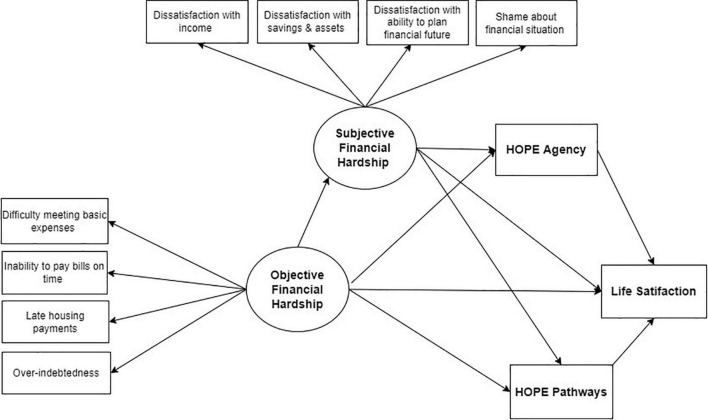
Hypothesized relationships between objective financial hardship (OFH), subjective financial hardship (SFH), hope agency, hope pathways, and life satisfaction (LS).

This study seeks to add to the research base on mechanisms of action of the relationship between financial hardship and LS. We examine whether SFH – measured as dissatisfaction with and shame about one’s financial situation – mediates the relationship between OFH and LS in an ethno-racially diverse sample of adults with psychiatric diagnoses who are unemployed or underemployed. We especially examine the role of hope, and its agency and pathways dimensions, in mediating the effect of SFH on LS. Figure 1 presents our conceptualized framework and the hypothesized relationships between objective and SFH, hope, and LS. These relationships have not been sufficiently examined in the general population, and to our knowledge have never been studied before among people with psychiatric diagnoses. We leverage cross-sectional data on a sample of adults with psychiatric diagnoses engaged in two peer-run employment programs, largely dependent on social security disability and welfare programs as main sources of income, and most of whom live with incomes below poverty. By elucidating the mechanisms through which financial hardship erodes subjective wellbeing in this population, we seek to inform the development of interventions for empowering unemployed and underemployed individuals with psychiatric conditions to improve their objective financial situation, buffer the impact of financial difficulties on their mental wellbeing, and ultimately avert further decline in their socio-economic and mental health condition.

## Materials and Methods

### Data

Data were collected from a convenience sample of 215 individuals with a psychiatric diagnosis who participated in two peer-run employment programs in New York City and upstate New York at the time of interview. Both programs provide supports free of charge to individuals who are not engaged in paid work or who are seeking new or additional employment. These supports include employment goal setting, résumé writing, interview coaching, transition to employment, and ongoing emotional assistance. These are provided by people with lived experience of psychiatric diagnoses and recovery with formal training as peer specialists. Both programs are housed in non-profit peer-run organizations also providing other peer supports, such as advocacy, community integration, and supported housing.

### Recruitment Procedures

Program staff invited recipients of employment services in 2015–2016 to participate in a financial and emotional wellness survey through face-to-face contacts, flyers, telephone, and mailed letters. All individuals who were active program participants during the survey period were invited to participate (*N* = 235). Successful interviews were completed with 215 individuals. Hence, the survey response rate was approximately 91.5%. Inclusion criteria were current participation in employment supports and being of working age (18–64). All survey respondents had a documented psychiatric diagnosis, as required for participation in both employment programs. Exclusion criteria included active florid psychosis or acute emotional distress at the time of consent, and difficulty understanding survey procedures, as assessed by program staff.

### Interviews and Interviewers

Structured interviews lasting 45–60 min were conducted by peer providers and managers of peer-run programs. The research team decided that program staff known to participants would be most effective in engaging potential research participants, developing trust, and collecting valid data about financial matters. Interviewers received 6 h of training by the first author (OJ-S). OJ-S led debriefing sessions with interviewers after the completion of the first and fifth interviews to provide additional coaching. All surveys were reviewed contemporaneously for completeness by the first author and the project’s lead research assistant.

### Ethics

The study was approved by the New York State Psychiatric Institute (NYSPI) Institutional Review Board as the IRB of record, which also approved a waiver of written documentation of consent. Before participating in study procedures, research participants were provided with a consent information sheet. After asking questions about study procedures, participants gave verbal consent. They received $30 USD in cash compensation and travel fare for completion of the interview. Completed questionnaires were de-identified before being transmitted to researchers in person or via certified mail.

### Measures

#### Indicators of Objective Financial Hardship

Objective financial hardship was observed using four items assessing the presence of different types of financial hardships: difficulty meeting basic expenses, inability to pay bills on time, housing unaffordability, and over-indebtedness. The items are: (1) “In a typical month, how difficult is it for you to cover your expenses and pay all your bills? (1, Not at all difficult; 2, Somewhat difficult; 3, Very difficult) (*difficulty meeting basic expenses*); (2) “I pay my bills on time” (1, Completely disagree; 2, Disagree; 3, Neither agree nor disagree; 4, Agree; 5, Completely agree) (*inability to pay bills on time*); (3) “How many times have you been late with your rent or mortgage payments in the last 2 years? (1, Never; 2, Once; 3, More than once) (*housing unaffordability*); (4) “I have too much debt right now” (1 = “strongly disagree” to 7 = “strongly agree”) (*over-indebtedness*). These items were obtained from the FINRA Financial Capability Study (items 1, 2, 3) (74) and the OECD Financial Literacy Survey (item 4) (75), two well-established national and international surveys of financial capability. Item 2 was reverse coded so that higher values indicate greater hardship.

#### Indicators of Subjective Financial Hardship

Subjective Financial Hardship was observed utilizing four items measuring respondents’ current dissatisfaction with their ability to meet their needs, savings/assets, ability to plan financial future, and feelings of shame about their current financial situation. The items are: (1) “How satisfied are you with how much money you have available every month to meet your needs?” (2) “How satisfied are you with how much savings you have and the number of things of monetary value you own?” (3) “How satisfied are you with your ability to plan your financial future?” and (4) “I feel ashamed about how bad my financial situation is.” Each item was assessed by a 5-point Likert scale. For items 1–3, response categories ranged from completely dissatisfied to completely satisfied; for item 4, from strongly agree to strongly disagree. Items were reverse coded so that higher values indicate greater subjective hardship. Items 1–3 were created to capture dissatisfaction with financial stability (ability to meet basic needs) and financial security (ability to build savings/assets and plan financial future), important domains in the financial wellness framework for people with psychiatric diagnoses proposed by Jiménez-Solomon and colleagues (76). The fourth item was developed to assess financial shame, a documented experience in response to financial hardship and poverty (61, 62). The research team drafted items with input from nine peer specialists (people in recovery from psychiatric diagnoses trained in peer-support principles and techniques) who provided employment supports at each site. The research team crafted eight items and reviewed them with peer specialists for understandability. Two items were drafted to assess each of four domains of SFH (i.e., dissatisfaction with their ability to meet needs, savings/assets, ability to plan financial future, and feelings of shame about their current financial situation). The research team then conducted a small pilot study with ten individuals with psychiatric diagnoses, and respondents provided their input via debriefing interviews. After the pilot, four items (one per domain) were selected based on comprehension and relevance and further edited before final survey implementation.

#### Adult Hope Scale

Developed based on Snyder’s hope theory, the adult hope scale is a validated 8-item measure of trait/dispositional hopeful thinking with adequate internal consistency (α = 0.74–0.88), test–retest reliability (68), and concurrent and discriminant validity (77). Factor analyses consistently support a two-factor model and an overarching construct of hope (78), which can be operationalized as a single composite or two subfactors, *agency* and *pathways*, following Snyder’s cognitive model of hope. In our sample, internal consistency for the single hope (α = 0.84), agency (α = 0.79) and pathways scales (α = 0.76) were adequate.

#### Hope Agency Subscale

Agency was assessed using four items tapping the perceived ability to achieve one’s life goals. Respondents indicated the extent to which they agreed with each of four statements on an 8-point Likert scale ranging from 0 (*definitely false*) to 7 (*definitely true*). The statements included: (1) “I energetically pursue my goals;” (2) “I meet the goals that I set for myself;” (3) “My past experiences have prepared me well for my future;” and (4) “I’ve been pretty successful in life.” Items were summed (range 0–28) so that higher values indicate greater agency.

**TABLE 1:** Demographic and clinical characteristics of the sample of people with psychiatric diagnoses in employment programs (*N* = 215).

	Total sample (*N* = 215)	
	*N*	%/Mean (SD)
Age	215	44.51 (11.58)
**Gender**		
Female	91	42.33
Male	124	57.67
**Ethno-racial identification^a^**		
Black/African American, non-Hispanic	91	42.72
White, non-Hispanic	65	30.52
Hispanic	34	15.96
Other	23	10.80
**Highest education completed**		
Did not complete high school	55	25.58
High school graduate/equivalent	94	43.72
More than high school	66	30.70
**Marital status/Living arrangement^(1)^**		
Married/Living with partner or spouse	28	13.08
Single never married	139	64.95
Separated, widowed, or divorced	47	21.96
**Employment status**		
Currently in paid employment/self-employed	36	16.74
Not in paid employment	179	83.26
Ever worked before^(2)^	171	95.55
**Last paid work^(3)^**		
Less than 1 year	81	46.29
At least 1 year, less than 3 years	27	15.43
3 years or more	67	38.29
**Average hours of work per week^(4)^**		
<30	25	69.44
≥30	11	30.56
**Individual annual income^b^**		
≤$5,885	62	28.83
5,885 up to $11,770	109	50.69
≥$11,770	44	20.47
**Household income^c(5)^**		
Less than FPL for household size	118	68.21
At least FPL for household size	55	31.79
**Main source of income**		
Salaries or wages	32	15.53
SSI	69	33.50
SSDI	57	27.67
TANF	29	14.08
Other	19	9.22
**Psychiatric diagnosis^d^**		
Schizophrenia spectrum	58	26.98
Bipolar spectrum	49	22.79
Depressive, anxiety trauma-related, or obsessive-compulsive	95	44.19
Other	13	6.05
12-month psychiatric hospitalization or ER visit^(1)^	59	27.57
**New York region**		
Downstate	96	44.65
Upstate	119	55.35

*^a^‘Other’ includes those reporting multiple racial groups, Alaska Native, Other Pacific Islander, Asian Indian, Chinese, Unspecified Other, West Indian, and Don’t Know.*

*^b^Income brackets represent Federal Poverty Limit (FPL) for a single-individual household at the time of data collection, at <50%, 50% to <100%, and ≥100% of FPL, respectively.*

*^c^Household income FPL based on self-reported household size with reference to appropriate FPL value. N = 42 individuals in our sample (19.5%) did not know or preferred not to answer this question.*

*^d^Schizophrenia-spectrum diagnosis includes schizophrenia and schizoaffective disorders. Depressive, anxiety, trauma-related, and obsessive-compulsive disorders include major depressive disorders, anxiety disorders, obsessive-compulsive disorders, and posttraumatic stress disorders. Other disorders include attention-deficit hyperactivity disorder, personality disorders, learning disabilities, and refused to answer or did not know their diagnosis.*

*^(1)^n = 214 (missing data on 1 individual).*

*^(2)^n = 179 (individual reporting no current paid employment).*

*^(3)^n = 175 (individual reporting no current paid employment, missing data on four individuals).*

*^(4)^n = 36 (individuals reporting current paid employment).*

*^(5)^n = 173 (missing data on 42 individuals).*

#### Hope Pathways Subscale

Pathways was assessed as the perceived capability of finding or activating routes to achieving one’s life goals. Respondents described their level of agreement with the following four statements: “I can think of many ways to get out of a jam,” “There are lots of ways around any problem,” “I can think of many ways to get the things in life that are important to me,” and “Even when others get discouraged, I know I can find a way to solve the problem.” Response options (0-7) range from 0 (*definitely false*) to 7 (*definitely true*). Items were summed (range 0-28) with higher values indicating a greater sense of pathways.

#### Satisfaction With Life Scale

We used Diener’s five-item measure to assess current global LS (79). Each item is assessed by a 7-point Likert scale 1–7 (*completely disagree – completely agree*). Each item is coded 0–6 and summed (range: 0–30), with higher values indicating greater LS. The five items are: (1) “In most ways my life is close to my ideal;” (2) “The conditions of my life are excellent;” (3) “I am satisfied with life;” (4) “So far, I have gotten the important things I want in life;” (5) “If I could live my life over, I would change almost nothing.” In our sample internal consistency for this scale was acceptable (α = 0.82).

#### Covariates

Structural Equation Modeling (SEM) in this study includes potential confounders, including binary gender (males = reference group), age (years), race/ethnicity (Black non-Hispanic, Hispanic, Other, White non-Hispanic [reference]), education (high school or above, below high school [reference], and self-reported diagnosis (1) Bipolar disorder, (2) Depressive, anxiety, trauma-related, and obsessive-compulsive disorders and other diagnoses [hereafter, “Other diagnoses”], (3) Schizophrenia and psychotic disorders [reference]), employment status (currently engaged in paid employment or self-employment, not engaged in paid employment or self-employment [reference]), income (individual income above Federal Poverty Line (FPL) for a household of one at the time of data collection, below poverty [reference]), receipt of Supplemental Security Income (SSI) or Social Security Disability Insurance (SSDI) (currently receiving SSI or SSDI, not receiving SSI/SSDI [reference]), and New York region (upstate, downstate [reference]).

### Analytic Approach

Confirmatory Factor Analysis (CFA) was employed to test measurement models for OFH and SFH indicators and SEM was used to test our mediation hypotheses. Models were estimated using Mplus software version 8.6 (80).

#### Confirmatory Factor Analysis of Objective Financial Hardship and Subjective Financial Hardship

We hypothesized that eight indicators of financial hardship had two related but distinct underlying latent factors: OFH and SFH. A two-factor CFA model was fit specifying the OFH latent factor underlying four indicators (difficulty meeting basic needs, inability to pay bills on time, housing unaffordability, over-indebtedness) and the correlated SFH latent factor underlying four additional indicators (dissatisfaction with income, dissatisfaction with savings and assets, dissatisfaction with ability to plan one’s financial future, shame about one’s financial situation). Estimation using weighted least squares (WLSMV) was employed, which takes into account the ordered categorical Likert-response nature of the items. Standardized coefficients for factor loadings were inspected to examine how strongly each item loaded within each factor. A one-factor CFA model underlying all eight items was also fit and compared. Absolute cut-offs indicating “adequate” or “good” fit for common CFA fit statistics (e.g., RMSEA < 0.08, CFI > 0.90) have been widely criticized (81, 82). We report them but focus on comparing them between models noting that a model with smaller RMSEA and larger CFI than another model indicates better fit (83). Factor loadings and fit statistics supported our hypothesis that OFH and SFH are best represented as two distinct factors rather than one (see Results section), hence our focus on separate OFH and SFH constructs in the mediation models below.

#### Testing Mediation Hypotheses

Structural Equation Modeling was used to examine, through increasingly complex models, three mediation hypotheses based on the theoretical time-order assumption that OFH precedes SFH, which precedes hope, which precedes LS. The three hypotheses are: (1) SFH mediates the effect of OFH on LS; (2) overall hope mediates the relationship between SFH and LS; and (3) hope agency and hope pathways each separately mediates the relationship between SFH and LS. To control for potential confounders, SEM models included gender, age, ethno-racial identification, educational level, self-reported diagnosis, employment status, income, SSI/SSDI receipt, and New York region, as predictors of each endogenous variable. Standardized path coefficients including total, direct, and indirect effects were estimated using WLSMV, which takes into account the ordered categorical Likert-response nature of all items, and tested using bootstrapping (with 1000 bootstrapped samples) to obtain standard errors and associated inference *p*-values. Missing data in WLSMV estimation is handled using pairwise present observations when forming the polychoric correlations so that individuals are not deleted if they have only partial missing observations. Nonetheless, approximately 6% of respondents were excluded from the final analytic sample (*N* = 202) due to missing data.

#### Effects of Covariates

Structural equation models also estimated standardized coefficients testing the independent effect of all sociodemographic and clinical predictors on key outcomes: OFH, SFH, overall hope, hope agency, hope pathways, and LS. For binary dummy variable predictors, the coefficients are standardized only for the outcome, not the predictor; hence, they represent the standardized differences in the outcome for different categories of predictor compared to the reference group, controlling for all other predictors. For instance, participants self-identifying as Black/African Americans reported on average a 0.39 SD higher LS than non-Hispanic Whites, while individuals self-identifying as Hispanics endorsed a 0.32 SD higher LS than non-Hispanic Whites (Table 4, Model 1).

## Results

### Sociodemographic Characteristics of Respondents

Table 1 summarizes the sociodemographic and clinical characteristics of our sample. Respondents had an average age of 44.4 years (SD = 11.6) and a majority identified as male (58%). The sample was racially and ethnically diverse, with 43% identifying as non-Hispanic Black/African American, 30% as non-Hispanic White, 16% as Hispanic, and 11% as another ethno-racial group (“Other”). Most reported low levels of education: ∼70% had a high school education or lower and a quarter of respondents reported less than high school. Over 80% were not employed, which is expected since respondents were recruited thought employment programs for individuals with psychiatric diagnoses, but is also consistent with the 83% estimated proportion of adults in the New York State public mental health system who are not employed (84). Of those who reported paid work, 69% indicated that they worked less than 30 hour per week, suggesting substantial underemployment. Among those employed at time of interview, almost all reported a prior work history (96%); close to half (46%) had not worked for under a year, 15% for 1–3 years, and 38% for over three years. The large proportion of individuals with past paid employment suggests the cyclical nature of un/employment in this population.

The sample’s overall low socio-economic status seems to be confirmed by their reported incomes. Four out of five (80%) reported individual annual incomes below the FPL for a household of one, with over a quarter reporting incomes equivalent to deep poverty (below 50% of FPL). Similarly, over two-thirds (68%) reported living in households with incomes below FPL. It is important to note that the household income estimate may not be representative of the full sample, since 42 respondents (20%) did not know this information or preferred not to answer. Consistent with large proportions of individuals with very low incomes, most respondents (75%) said their main source of income came from a social security or welfare program. For 61%, their main source of income came from cash benefits from one of two United States social security programs for people with disabilities, SSI and SSDI. For an additional 14%, their main source of income was from Temporary Assistance for Needy Families (TANF), a United States welfare program for individuals with no or very low income who are not or not-yet eligible for SSI or SSDI. Most respondents were single or living alone, with only 13% married or living with a partner. In terms of clinical characteristics, respondents self-reported diverse diagnoses: 27% in the schizophrenia spectrum, 23% in the bipolar spectrum, and 50% other diagnoses. Over one-quarter reported a psychiatric emergency room visit or hospitalization in the prior year, suggesting that a meaningful proportion of our sample had recently experienced high levels of psychiatric distress.

Table 2 summarizes the mean scores for the independent and dependent variables and the hypothesized mediators in our conceptual model. Most mean scores for the hypothesized indicators of OFH were slightly above the midpoint in the range of possible values, suggesting a substantial level of financial hardship. Most SFH indicators had mean scores that were noticeably above the midpoint (3.2–3.8 on a scale of 1–5), also suggesting that the average respondent endorsed moderate-to-high levels of SFH.

**TABLE 2:** Descriptive statistics for independent and dependent variables and hypothesized mediators in a sample of people with psychiatric diagnoses in employment programs (*N* = 215).

Variable	*N*	Range	Mean	SD
**Objective financial hardship (OFH) indicators**				
Difficulty meeting basic expenses	205	1–3	2.10	0.78
Inability to pay bills on time	215	1–5	2.37	1.19
Housing unaffordability	203	1–3	1.65	0.89
Over-indebtedness	211	1–7	4.06	2.44
**Subjective financial hardship (SFH) indicators**				
Dissatisfaction with income to meet needs	215	1–5	3.79	1.17
Dissatisfaction with savings and assets	215	1–5	3.89	1.11
Dissatisfaction with ability to plan financial future	215	1–5	3.42	1.25
Shame about financial situation	215	1–5	3.24	1.34
Adult Hope Scale	206	0–56	37.75	11.09
Hope Agency Subscale	210	0–28	18.33	6.40
Hope Pathways Subscale	211	0–28	19.46	5.89
Life satisfaction (LS)	213	0–30	13.77	7.48

The mean LS score suggests a below-average level of subjective wellbeing relative to the general population. Prior studies have found average LS scores in adults of 18.6 to 22.9 (85). The average LS score in our sample (13.77) falls within the below-average LS range (10–14) on a scale of 0 to 30. Although the mean score for overall hope is in the upper range of the scale, it also appears to be lower than the average score of 40 reported in the general population (86). These findings are consistent with research showing significantly lower hope indices in clinical samples than in the general population (77).

#### Confirmatory Factor Analysis for Objective Financial Hardship/Subjective Financial Hardship

We hypothesized that eight indicators of financial hardship had two related but distinct underlying factors: OFH and SFH. The correlation between OFH and SFH was statistically significant and high (*r* = 0.719; *p* = 0.000). Nevertheless, the CFA for the two-factor model showed improved fit (RMSEA = 0.088; CFI = 0.969) over a single factor model (RMSEA = 0.129; CFI = 0.931). In our two-factor model, the four hypothesized indicators of OFH demonstrated strong and highly significant loadings (ranging from 0.65 to 0.73). Similarly, the four indicators of SFH had moderate-to-strong and highly significant loadings (ranging from 0.45 to 0.91).

### Structural Equation Models

#### Model 1

**FIGURE 2:**
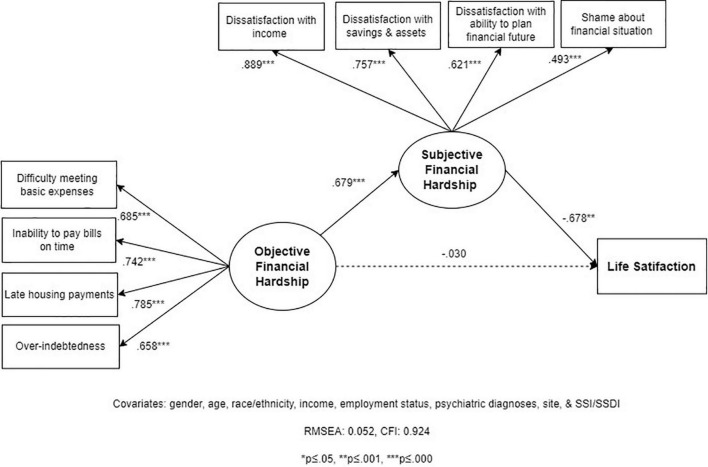
Structural Equation Model 1 (SEM 1): relationships between objective financial hardship (OFH), subjective financial hardship (SFH), and life satisfaction (LS; *N* = 202).

Structural Equation Modeling-1 tested whether SFH mediates the association between OFH and LS (Hypothesis 1). The model has acceptable fit statistics (RMSEA = 0.052; CFI = 0.924). After accounting for covariates, OFH has a strong effect on SFH (standardized *B* = 0.679, *p* = 0.000), which in turn has a strong and negative effect on LS (standardized *B* = –0.678; *p* = 0.000) (see Figure 2). The total, direct, and indirect estimates help quantify the extent to which SFH mediates the relationship between OFH and LS (see Table 3). Estimates indicate that while OFH has a moderate-to-strong total effect on LS (*B* = –0.491; *p* = 0.000), 94% of that effect is mediated by SFH, with a standardized indirect effect of 0.461 (*p* = 0.004).

**TABLE 3:** Total, direct, and indirect effects for Structural Equation Models 1, 2, and 3 (*N* = 202).

Paths	Total effect	Direct effect	Indirect effect	Percentage of total effect
**Model 1**				
OFH → SFH → LS	–0.491***	–0.030	–0.461**	93.89%
**Model 2**				
OFH → SFH → HOPE → LS (All paths)	–0.491***	0.008	–0.499***	100.00%
OFH → SFH → LS	–0.491***	0.008	–0.400**	81.15%
OFH → HOPE → LS	–0.491***	0.008	–0.039	–
OFH → SFH → HOPE → LS	–0.491***	0.008	–0.060*	12.22%
OFH → SFH → HOPE	–0.307**	–0.121	–0.186^†^	60.59%
SFH → HOPE → LS	–0.678***	–0.590***	–0.088*	12.99%
**Model 3**				
OFH → SFH → AGENCY + PATHWAYS → LS (All paths)	–0.491***	0.011	–0.502***	100.00%
OFH → SFH → AGENCY → LS	–0.491***	0.011	–0.091*	18.54%
OFH → AGENCY → LS	–0.491***	0.011	–0.043	–
OFH SFH→ PATHWAYS → LS	–0.491***	0.011	0.001	–
OFH → PATHWAYS → LS	–0.491***	0.011	0.001	–
OFH → SFH → LS	–0.491***	0.011	–0.367**	74.75%
OFH → SFH → AGENCY	–0.364**	–0.116	–0.248*	68.13%
OFH → SFH → PATHWAYS	–0.198	–0.129	–0.068	–
SFH → AGENCY → LS	–0.677***	–0.542***	–0.135**	19.94%
SFH → PATHWAYS → LS	–0.658***	–0.542***	–0.001	–

OFH, objective financial hardship; SFH, subjective financial hardship; LS, life satisfaction. ^†^p ≤ 0.10; *p ≤ 0.05; **p ≤ 0.01; ***p ≤ 0.001.

**TABLE 4:** Standardized coefficients for predictors and socio-demographic covariates (*N* = 202).

	Model 1 (*n* = 202)			Model 2 (*n* = 202)				Model 3 (*n* = 202)				
	OFH	SFH	LS	OFH	SFH	Hope	LS	OFH	SFH	Hope agency	Hope pathways	LS
**Predictors**												
OFH		0.679***	–0.030		0.678***	–0.121	0.008		0.677***	–0.116	–0.129	0.011
SFH	–	–	–0.678***	–	–	–0.274*	–0.590***	–	–	–0.367*	0.101	–0.542***
Hope	–	–	–	–	–	–	0.321***					
Hope agency	–	–	–	–	–	–	–	–	–	–	–	0.367***
Hope pathways	–	–	–	–	–	–	–	–	–	–	–	0.008
**Covariates**												
Black/African American, non-Hispanic	0.261	–0.249	0.390**	0.260	–0.252	0.439*	0.247^†^	0.260	–0.255	0.469**	0.384*	0.211
Hispanic	–0.082	–0.280	0.317^†^	–0.082	–0.285	0.517**	0.147	–0.082	–0.287	0.434*	0.472*	0.148
Other race/ethnicity	0.151	0.169	0.649**	0.149	0.162	0.532*	0.473*	0.148	0.158	0.419	0.527*	0.482*
Bipolar	0.692***	0.189	0.150	0.694***	0.192	0.184	0.093	0.696***	0.193	0.225	0.144	0.070
Other diagnoses	0.696***	–0.112	0.023	0.697***	–0.112	0.224	–0.048	0.698***	–0.112	0.307^†^	0.073	–0.089
Female	0.195	0.038	0.171	0.192	0.040	–0.108	0.206	0.191	0.042	–0.040	–0.145	0.188
Age	0.105	0.110	0.071	0.104	0.111	0.037	0.059	0.104	0.111	–0.004	0.087	0.072
High school or higher	0.270	0.145	–0.097	0.267	0.148	–0.138	–0.052	0.266	0.148	–0.009	–0.212	–0.092
Employed	–0.171	–0.124	–0.041	–0.171	–0.121	–0.144	–0.008	–0.170	–0.120	–0.170	–0.001	–0.025
Above poverty	–0.038	–0.318*	–0.214^†^	–0.037	–0.317*	–0.021	–0.206^†^	–0.037	–0.317*	–0.017	–0.013	–0.206^†^
SSI or SSDI receipt	–0.445*	–0.103	0.040	–0.446*	–0.099	0.032	0.032	–0.446*	–0.097	0.133	–0.140	–0.004
Upstate	0.425**	–0.192	0.036	0.425**	–0.192	0.142	–0.010	0.425**	–0.191	0.192	0.108	–0.035
**Model fit statistics**												
RMSEA CFI TLI	0.052 0.924 0.887			0.054 0.917 0.867				0.054 0.915 0.854				

OFH, objective financial hardship; SFH, subjective financial hardship; LS, life satisfaction. ^†^p ≤ 0.10; *p ≤ 0.05; **p ≤ 0.01; ***p ≤ 0.001.

#### Model 2

**FIGURE 3:**
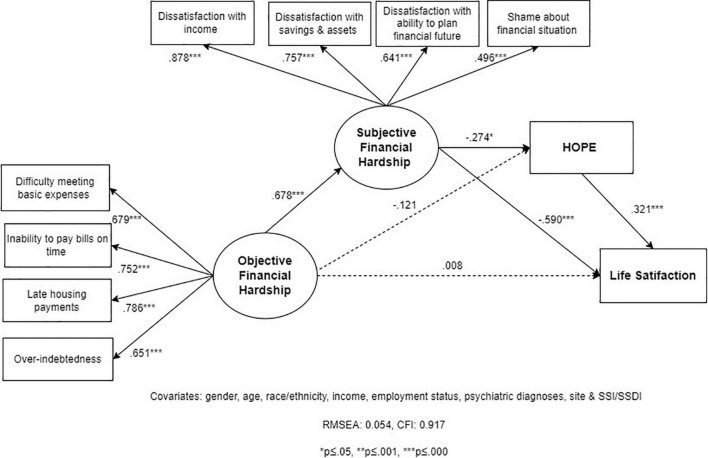
Structural Equation Model 2 (SEM 2): relationship between objective financial hardship (OFH), subjective financial hardship (SFH), hope, and life satisfaction (LS; *N* = 202).

We hypothesized that hope mediates the effect of OFH and SFH on LS (Figure 3). This SEM demonstrated acceptable fit statistics (RMSEA = 0.054; CFI = 0.917). As in Model 1, OFH has a strong direct effect on SFH (0.678; *p* = 0.000) but did not have a significant direct effect on hope (–0.121, *p* = 0.458) after accounting for the mediator SFH. SFH shows a strong direct effect on lower LS (–0.590; *p* = 0.000) that is not mediated by hope. The associations between SFH and hope (–0.274; *p* = 0.049), and hope and LS (–0.321; *p* = 0.000), respectively, are small.

Total, direct, and indirect effects indicate that all (100%) of the overall total effect of OFH on LS is mediated by SFH and hope (Table 3). Most of this indirect effect is due to paths being specifically mediated through SFH. SFH alone mediates 81% of the total effect between OFH and LS, with a moderate indirect effect of –0.40 (*p* = 0.002) (Table 3). Furthermore, the indirect effect through the chained path from OFH → SFH → hope → LS is small and borderline significant (–0.060; *p* = 0.047). We find that OFH has a weak-to-moderate total effect on hope (–0.307; *p* = 0.006) and that SFH mediates close to 60% of this effect, though the indirect effect does not reach significance (–0.186; *p* = 0.074). Similarly, hope mediates the effect of SFH on LS, but this indirect effect is very weak (–0.088; *p* = 0.032) and only represents 13% of the association between SFH and LS.

#### Model 3

**FIGURE 4:**
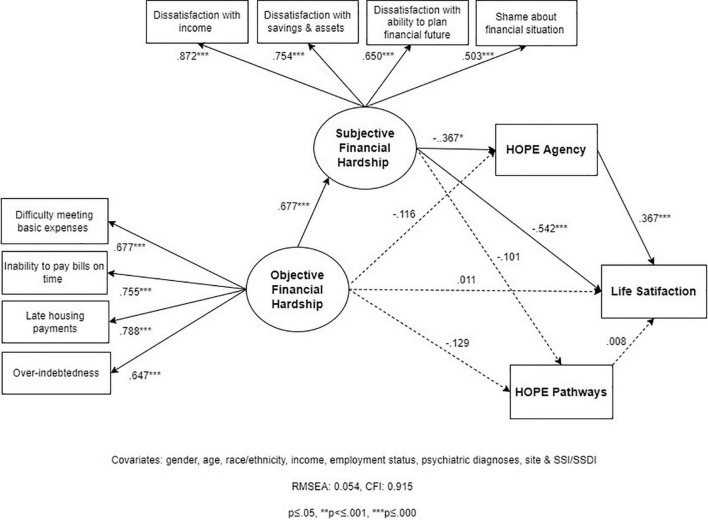
Structural Equation Model 3 (SEM 3): relationships between objective financial hardship (OFH), subjective financial hardship (SFH), hope agency, hope pathways, and life satisfaction (LS; *N* = 202).

Based on prior research and our conceptual framework, we hypothesized that both hope agency and hope pathways mediate the relationship between SFH and LS (Figure 1). SEM-3 has overall acceptable fit statistics (RMSEA = 0.054; CFI = 0.915). In summary, the findings of model 3 indicate that the relationship between SFH and LS is partially mediated by hope agency, but not hope pathways (Figure 4). Similarly, SFH partially mediates the effect of OFH on hope agency, but not hope pathways. We describe below the specific findings that support this conclusion.

As in SEM-2, the total effect of OFH on LS is fully mediated (100%) by SFH and the hope measures, and most of that effect (74.8%) is from SFH alone (Table 3). The associations between SFH and hope agency (–0.367; *p* = 0.017), and hope agency and LS (–0.367; *p* = 0.000), respectively, are weak-to-moderate. On the other hand, the associations between SFH and hope pathways (–0.129; *p* = 0.494), and hope pathways and LS (0.008; *p* = 0.912), respectively, are very weak and non-significant. As a result, the portion of the mediated effect from OFH to LS that goes through hope is only going through hope agency (18.5%) and not through hope pathways (0%). Similarly, hope agency mediates the effect of SFH on LS, but this indirect effect is also weak (–0.135; *p* = 0.013) and represents only about 20% of the total effect of SFH on LS.

Although these findings indicate that hope agency is only a partial mediator, the indirect effect of SFH on LS through hope agency (in Model 3) is 1.5 times greater than via the overall hope measure (in Model 2) (20% vs. 13%). Taken together, these findings suggest that whatever role hope has as a mediator of the effect of SFH on LS, this mediating effect is mainly through its agency component.

### Effects of Sociodemographic and Clinical Covariates

This section summarizes our main findings on the association between socio-demographic covariates and key outcomes: OFH, SFH, hope, hope agency, hope pathways, and LS. Table 4 presents the standardized coefficients estimating the independent effect of all predictors in each of our three models.

#### Race/Ethnicity

Ethno-racial identification was not significantly associated with OFH or SFH after controlling for other covariates. Nevertheless, compared to non-Hispanic Whites, those identifying as Black/African American, Hispanic, and other minoritized ethno-racial identities reported significantly higher LS, overall hope, and hope pathways, net of the effect of OFH and SFH. This was also the case for hope agency for Black/African American and Hispanic individuals, but not for participants of other minoritized races/ethnicities. To contextualize the effect of minoritized ethno-racial identities, we estimate that, after adjusting for the effect of OFH and all other covariates, a one-SD higher SFH was associated with a 0.68 SD lower LS, while identifying as Black/African American, Hispanic, and other minoritized ethno-racial individuals was associated with 0.39, 0.32, and 0.65 SD higher LS, respectively (Model 1). These findings suggest that identifying as Black/African American, Hispanic, and other minoritized ethno-racial identities may partly or fully buffer the effect of small differences in OFH and SFH on hope and LS, depending on other covariates. It is noteworthy that in Models 2 and 3, which account for the mediating effect of hope and hope agency, the protective effect of Black or Hispanic ethno-racial identification on LS is noticeable smaller and less significant, in contrast to Model 1, suggesting that part of the buffering effect of minoritized ethno-racial identification may be through increased hope.

#### Psychiatric Diagnoses

Across all three models, individuals with bipolar disorder, and those with other diagnoses, reported higher OFH than those with schizophrenia-spectrum disorders (by ∼0.70), after adjusting for all other covariates. Diagnosis was not associated with any other mediator or outcome measure in Models 1–3.

#### Income

Income above poverty level was not significantly associated with OFH. Nevertheless, individuals with incomes above the poverty level reported on average 0.32 SD lower SFH than those with incomes below poverty. Above-poverty income was not associated with hope. However, contrary to our expectations, higher income was associated with lower LS (–0.21) across models, although coefficients for this covariate were only significant at *p* ≤ 0.10.

#### Supplemental Security Income or Social Security Disability Insurance Receipt

Receiving SSI or SSDI was associated with significantly lower (–0.45) OFH, after controlling for other covariates.

#### New York Region

Respondents in our upstate New York site reported significantly higher (0.43) OFH that their downstate counterparts, net of the effect of individual-level characteristics.

Other covariates did not have a statistically significant association with any of the outcome variables. Most notably, being employed did not have a statistically significant association with OFH, SFH, hope, or LS.

## Discussion

This study employed SEM to test, via CFA, measurement models for OFH and SFH in a sample of low-income, ethno-racially diverse, and predominantly unemployed or underemployed adults with psychiatric diagnoses. We hypothesized related but distinct, single factors for OFH and SFH. We also fit three full structural equation models. An initial model examined whether SFH mediates the association between OFH and LS. Two additional models were specified to estimate the extent to which overall hope, and its agency and pathways components, further meditate the effect of SFH on LS. Several conclusions can be drawn from the findings of the structural equation models.

First, confirmatory factor analyses supported our proposed scales for objective and subjective hardship. The hypothesized indicators of OFH and SFH loaded strongly within each factor, and a two-factor model demonstrated improved fit over a one-factor model. Our three full structural equation models provided additional evidence about a two-factor structure. All three models demonstrated acceptable fit and indicators loaded strongly within each factor. Taken together, these findings suggest that the indicators (a) difficulty meeting basic needs, (b) inability to pay bills on time, (c) housing unaffordability, and (d) over-indebtedness comprise a factor that is distinct from the subjective experience of financial hardship. Similarly, the proposed indicators (a) dissatisfaction with one’s ability to make ends meet, (b) with savings and assets, and (c) with the ability to plan a financial future, and (d) shame about one’s overall financial situation seem to be part of a dimension of financial hardship that is related but separate from more concrete indicators of one’s financial situation. These findings are consistent with the robust literature that distinguishes objective and subjective dimensions of the financial hardship experience (13, 87), and finds that indicators of satisfaction with and shame about one’s financial situation reflect the subjective experience of concrete financial hardships, such as difficulty meeting basic needs, housing insecurity, and indebtedness (13, 61–65, 88, 89). Future research should examine the validity and reliability of these measures, including their predictive validity in relation to subjective wellbeing and mental health.

A second conclusion from our findings is that while both OFH and SFH have strong effects on LS (0.49 and 0.68, respectively, on a standardized scale), after controlling for potential confounders, the effect of SFH is noticeably stronger. An increase of 1 SD in OFH is associated with a 0.49 SD decrease in LS, while a 1 SD increase in SFH is associated with a 0.68 SD decrease. These findings are consistent with research documenting stronger associations between LS and SFH indicators, relative to measures of OFH. In fact, the strength of the association between SFH and LS found in our study echoes magnitudes found in prior research (20, 21). For some individuals, experiencing OFH may not translate into lower LS. Their subjective experience of OFH may be buffered by a sense of social support, self-efficacy, and other internal and external protective factors. Thus, SFH may be closer in the causal pathway toward LS and mediate the effect of OFH on LS. This brings us to a third conclusion from our findings: SFH mediates almost all (94%) of the effect of OFH on LS. In models that integrate overall hope, hope agency, and hope pathways, SFH by itself still mediates most of the effect (at least 75%). These findings are consistent with extensive research indicating that SFH mediates a significant proportion of the effect of OFH indicators on LS. Lacking cash to meet basic needs, inability to pay bills, indebtedness, and overall financial hardship seem to create dissatisfaction with one’s financial situation, a sense of loss of financial control, feelings of threat, and shaming experiences, which, in turn, lower individuals’ sense of satisfaction with life (35, 59, 60, 63).

Our two-level mediation models, which incorporate SFH, overall hope, and the two hope components, support a fourth and important conclusion. Decreased overall hope partially mediates the effect of SFH on LS, but this indirect effect is modest (*B* = –0.09 SD) and represents only 13% of the total effect on LS. The indirect effect of SFH through hope agency is slightly larger but still modest (*B* = –0.14) and equivalent to only 20% of the effect. The indirect effect via hope pathways nears zero. Thus, SFH has a strong and independent effect on LS beyond its effect through hope. SFH alone mediates most of the effect of OFH on LS, with a moderate indirect effect between *B* = –0.37 and *B* = –0.40 depending on the model (representing 75–81% of the total effect of OFH). Our findings are consistent with recent literature indicating that hope only partially mediates the relationship between financial hardship and LS (63, 67). We are unaware of any previous study testing a two-level mediation model to examine the relationships between OFH and SFH, hope, and LS.

These findings also suggest that hope agency may be a more relevant hope component in the relationship between SFH and LS than hope pathways. Our two-level mediation model that estimates the disaggregated mediating effect of hope agency and pathways (Model 3) shows that the association between SFH and LS is partly mediated by hope agency, but not pathways. This finding is consistent with prior research showing the stronger mediating role of agency, relative to pathways, on the relationship between income and LS (66). More generally, prior research has found that agency is a stronger predictor of subjective wellbeing, relative to pathways, which suggests that the belief that one is able to achieve personal goals may be more instrumental to LS than the perception that there are routes for doing so (66, 71). This finding is also supported by research suggesting that agency, and not pathways, mediates the effect of positive affect on LS (90). Even in the face of multiple barriers to one’s goals (or the perception that there are few routes to achieving them), the belief that one is capable of pursuing and staying motivated to pursue these goals may still produce a greater sense of wellbeing (72). This may be an encouraging finding, as it suggests that it may be possible to foster subjective wellbeing by building a sense of agency, even if individuals lack a clear sense of the specific paths toward their goals.

Our models also provide exploratory evidence regarding the association between socio-demographic and clinical covariates and key outcomes. Most notably, we find no statistically significant differences in OFH or SFH across ethno-racial groups. This is unexpected given economic inequities across these groups at the population level (91, 92). In contrast, across models, identification as Black/African American, Hispanic, and other minoritized ethno-racial identities was associated with noticeably higher overall hope, agency, pathways, and LS, relative to non-Hispanic Whites, net of the effect of OFH and SFH and other covariates. Taken together, these findings suggest that minoritized ethno-racial identification may play an important role in buffering the effect of OFH and SFH on LS, which is consistent with prior research. Among Blacks/African Americans, for instance, a greater sense of self-worth, mastery, and family supports seem to buffer the effect of financial stress on psychological wellbeing (93).

The stronger sense of agency and pathways, and more positive evaluation of life, among Blacks/African Americans, Hispanics, and those identified with other minoritized ethno-racial groups may also result from ethno-racial/cultural differences in social support, faith, and salience of social comparison (94–97). For instance, Black individuals tend to report higher levels of religiously or spiritually inspired hope than non-Hispanic Whites (98). It is also possible that the role of social expectations or social comparisons may be stronger among non-Hispanic Whites than these two minoritized groups. For non-Hispanic Whites who grow up with expectations of middle-class living and economic advancement, the reality of a disabling condition and financial exclusion may present greater challenges to one’s goals and have a greater impact on overall LS (99).

Although research about the association between psychiatric diagnoses and financial hardship is scarce, our finding that individuals with schizophrenia-spectrum disorders report lower OFH than people with other conditions is unexpected. Since individuals with schizophrenia-spectrum diagnoses are more likely to experience long-term disability than people with other psychiatric diagnoses (100–102), they may be also more likely to experience unemployment, underemployment, dependency on disability benefits, and financial hardship (103, 104). Instead, in this study, people with schizophrenia-spectrum disorders report a *B* = 0.7 SD lower OFH than individuals with other diagnoses. It is possible that individuals with bipolar disorder may be especially vulnerable to experiencing financial instability because of their psychiatric conditions. For instance, psychological traits more common in bipolar disorder, such as compulsive spending, “comfort” purchasing, and inattention to financial matters, may contribute to increased financial difficulties in this population. Compulsive spending may increase indebtedness, while “comfort” purchases may misdirect limited funds and compromise individuals’ ability to meet basic needs (105–107). Nevertheless, this would not seem to explain why individuals with other diagnoses (e.g., major depression, anxiety disorders, personality disorders) also report more difficulties making ends meet, inability to make timely payments on bills and housing, and indebtedness.

A likely, but paradoxical, explanation for the lower OFH reported by respondents with schizophrenia-spectrum disorders, relative to other diagnoses, may be their higher likelihood of experiencing long-term disability. People with schizophrenia-spectrum diagnoses are more likely to depend on SSI/SSDI than people with other disorders. In our sample, for instance, people with schizophrenia-spectrum conditions were more likely to receive SSI or SSDI (90% vs. 58%, *X*^2^ = 19.20; *p* = 0.000) and less likely to receive TANF (18% vs. 31%, *X*^2^ = 3.74; *p* = 0.055) than those with other diagnoses. Although the cash benefits provided by SSI/SSDI are generally poverty-level, these social security disability programs offer a stable source of income to meet basic needs. TANF, a program that provides cash assistance to low-income families, is designed as temporary support, capped to a lifetime maximum of 60 months, and includes work requirements. For people with long-term disabilities, SSI/SSDI provide a source of more permanent income – a safety net that people with schizophrenia-spectrum disorders may be more likely to receive. A second, seemingly paradoxical, explanation for this relationship with lower OFH may involve the greater financial exclusion of individuals with schizophrenia-spectrum disorders. Our measure of OFH presupposes a minimum level of financial engagement, such as having rent payments, bills, and debts. It is possible that people with schizophrenia-spectrum disorders are less likely to endorse some of these indicators because they lack access to loans or credit cards, or because they do not have housing or utility bills that they are directly responsible for. It is also possible that study participants with these conditions may receive, on a long-term basis, community mental health services (e.g., case management) ensuring some of their basic needs. A sign of the financial exclusion of people with schizophrenia-spectrum disorders in our sample is their greater reliance on representative payees – individuals designated to receive SSA payments and make financial decisions on behalf of SSI/SSDI recipients. Among study participants with SSI/SSDI, individuals with schizophrenia-spectrum disorders were more likely to have a representative payee compared to those with other diagnoses (40% vs. 25%; *X*^2^ = 3.25; *p* = 0.07). Representative payees may ensure that an individual’s bills and basic needs are addressed. Individuals with a representative payee may also be less aware of their financial situation and thus underreport financial difficulties.

Our models indicate that employment status was not significantly associated with OFH, SFH, hope, or LS in our sample. Although initially surprising, given the extensive literature on the benefits of employment for subjective wellbeing (49, 108), the observed relationships must be understood in the context of the likely employment conditions and overall financial situation of this population. In our study, although paid employment was associated with significantly higher income relative to unemployment, employed participants’ income was still extremely low. All individuals who engaged in paid employment reported incomes below 200% FPL for a household of one (results not shown). In addition, over two-thirds of employed individuals reported only part-time employment. From an objective financial perspective, paid employment in this population may bring additional income but not be sufficient to lessen financial hardship. It is also likely that the types of jobs held by study participants may not provide the psychological benefits expected from employment, since underemployed individuals like those in our study tend not to report better LS than unemployed persons (54). Also notable was that the subsample of individuals with incomes above the poverty level did not report lower OFH, contrary to general-population findings of greater OFH among lower-income individuals (109). As in the case of employment/underemployment, this may be due to the limited range of incomes in our sample: even those reporting the highest incomes would be considered low-income by broader economic standards. However, study participants with incomes above the poverty level reported significantly lower SFH (about 30% lower than those with lower incomes). This suggests that, although slightly higher incomes may not affect OFH indicators, they may be enough to improve individuals’ satisfaction with their financial situation, possibly by lessening their finances-related shame and providing a more positive outlook on their financial future.

In summary, OFH was a stronger predictor of SFH, hope, and LS than individual income or employment status. Unlike income and employment status, OFH had a strong effect on SFH, a moderate-to-strong effect on LS, and a weak-to-moderate effect on overall hope. Taken together, these findings are consistent with research indicating that financial hardship may be a stronger predictor of subjective wellbeing than income, and that low income may lose its independent effect on LS after accounting for financial hardship (20, 21).

Across all models, the New York upstate site reported significantly higher OFH than the downstate New York site. This finding may reflect the impact of ecological factors associated with the sites’ geographic areas and where participants were likely to reside. The upstate site is located in a mid-size city that is less economically vibrant than our downstate site and surrounded by zip codes where ∼30% of residents live in poverty. Most upstate participants were Black/Hispanic and probably more likely to reside in racially segregated and low-resource neighborhoods. By contrast, our downstate site is in a large, economically vibrant metropolis, surrounded by zip codes with poverty levels below 10%, and more ethnically diverse. It is likely that each geographic area provides different access to economic opportunities and resources that operate through ecological and individual-level factors not accounted for in our models. As such, coefficients for our site variables may be a proxy for the effect of ecological-level factors.

### Research Contributions and Implications

Study findings contribute to the literature on financial hardship, hope, and LS among unemployed and underemployed individuals with psychiatric diagnoses in several ways. First, this study may be the first to provide preliminary evidence of the relationships between OFH, SFH, hope, and LS in this population. Future research should clarify the mechanisms through which OFH erodes the subjective wellbeing of people with psychiatric diagnoses. Information on mechanisms of action would inform the development of effective interventions in tandem with supported employment supports to foster the financial wellbeing in this population, buffer the impact of financial difficulties on their mental wellbeing, and ultimately avert further mental health decline. A second contribution involves the finding of a potentially buffering effect that ethno-racial self-identification may play on the impact of financial hardship on LS. Future research should examine the relationship between racialized identities, OFH, SFH, hope and LS to inform interventions for communities likely to experience the compounded effect of poverty, racism, and social exclusion.

Thirdly, this study contributes more generally to research on the mediating role of subjective experience on the relationship between objective indicators of financial hardship and LS. While research has consistently shown that subjective measures of financial wellbeing have a stronger relationship with LS than objective measures (13, 55, 56), some studies seem to take this to mean that OFH is irrelevant. For instance, in their study about financial hardship, hope, and psychological wellbeing, Frankham and colleagues seem to conclude that OFH is not in the causal pathway because its association with psychological wellbeing is no longer significant after controlling for SFH (63). Consequently, their study excludes OFH from the mediation analyses, missing the opportunity to simultaneously explore the relationships between OFH and SFH, hope, and wellbeing. In our view, objective and subjective dimensions of financial hardship should not be considered as opposing or competing explanatory factors. Instead, both objective and subjective measures should be integrated within the same causal models. Future research should leverage theories and methodologies to conduct more conclusive empirical research in this area.

### Implications for Practice

Our findings suggest that economic empowerment interventions that are effective at reducing OFH and SFH among individuals with psychiatric diagnoses may meaningfully improve their LS. Financial empowerment interventions should integrate specific strategies to lessen OFH across specific areas, such as difficulty meeting basic needs or paying bills on time, housing unaffordability, and over-indebtedness. This should include support accessing and navigating financial wellness resources that are often unfamiliar to people with psychiatric conditions and their providers, such as the Earned Income Tax Credit, the Child Tax Credit, free financial counseling services, free tax preparation assistance, housing eviction prevention programs, and a host of work incentives for individuals receiving SSI or SSDI. Supported employment programs may be an opportune context in which to integrate economic empowerment interventions, as individuals engaged in those programs may be especially motivated to address objective and subjective financial stressors. Financial empowerment interventions should also incorporate strategies to buffer the stress associated with their financial difficulties. For instance, interventions should build shame-resilience skills around financial difficulties, such as recognizing when one is ashamed, identifying what triggers this feeling, and seeking empathy and support (110, 111). These strategies may include activities that build financial shame resilience led by trained peer specialists, whose lived experience provides a unique source of empathy and trust-building (112, 113).

Strategies to concurrently reduce OFH and SFH while fostering hope may also be important in promoting subjective wellbeing. This study shows that hope, and specifically its agency component, has a weak-to-moderate association with LS, even after accounting for the effect of OFH and SFH. By promoting hope, interventions may help buffer the impact of OFH and SFH and foster the subjective wellbeing of unemployed and underemployed individuals with psychiatric diagnoses. This finding may have important implications for practice in psychiatric rehabilitation and peer services. Hope is widely recognized as an important predictor of mental health recovery (114) and research has demonstrated that hope is the most consistent evidence-based outcome of peer services (115–117). Nevertheless, to promote hope most effectively, psychiatric and peer services must clearly define this construct, operationalize its components, and devise sensible strategies that tap into the strengths of rehabilitation and peer-led interventions.

To build agency, for instance, programs could focus on activities that increase self-efficacy and optimism. Self-efficacy, the belief that one can take the action required to achieve a desired outcome (118), can be developed over time and influenced through various experiences, such as personal successes, observation of others’ behaviors and successes, and the encouragement of others (119). Interventions can also build optimism – defined as an individual’s thoughts and feelings about a positive future – to garner the many benefits of optimism, including higher levels of subjective well-being, increased health, and greater success in multiple domains of life (114). Both self-efficacy and optimism have been found to positively impact LS (115, 116). Strategies to build self-efficacy, optimism, and overall agency may include sharing personal stories of financial empowerment, supporting individuals to develop concrete financial wellness action plans, and fostering mutual support. Such strategies may not only buffer the impact of OFH and SFH on subjective wellbeing, but also improve the engagement of individuals in their financial empowerment journeys.

Our findings also add to growing evidence about the role of financial hardship as a social determinant of mental health (117, 120). As such, our findings support the case for policy-level interventions aimed at reducing financial hardship, such as expanding access to affordable financial services, tax credits, housing and food supports, and financial counseling (121, 122).

Addressing financial hardship as a social determinant of mental health and wellbeing requires that mental health policy work in tandem with economic policy. Mental health policy ought to expand service structures and funding to support economic empowerment interventions, and economic policy should include specific features to engage and respond to the needs of individuals with psychiatric diagnoses.

Cross-sectorial collaborations may be especially effective at tackling financial hardship as a root cause of poor mental health. In the United States, for instance, a growing number of municipal governments, with the support of the coalition Cities for Financial Empowerment, fund free financial counseling services for their residents as an economic development strategy. Local government initiatives to improve mental health and wellbeing may be most effective if implemented in coordination with such economic empowerment efforts. Other examples of cross-sectorial collaborations include policy initiatives in the United Kingdom that would provide a debt repayment moratorium – ‘breathing space’ – for individuals experiencing a mental health crisis and regulate debt collection practices to minimize their psychological impact (123, 124). Future research should examine the feasibility and efficacy of these types of initiatives.

### Limitations and Future Directions

Our study has several limitations. The small sample size may have limited our ability to detect effects and biased our estimates. Therefore, our findings should be regarded as exploratory, and null effects should be interpreted with caution. The self-reported nature of OFH indicators may have introduced a level of measurement error. Hence, our findings should be regarded as approximate. Nevertheless, studies have found that self-reported measures of financial hardship can adequately predict observed financial indicators (e.g., actual debt amounts, payments) (125), and that measurement error associated with self-reported indicators does not explain differences in financial hardship across groups (126).

Our data are cross-sectional, which prevented us from establishing temporal relationships between variables in our SEM estimates and to employ statistical methods to approximate causality, such as fixed effects. Future research should examine the causal relationships between financial hardship, hope, and LS over time with larger samples. It would be especially important to better examine the direction of causality since research has documented the bidirectional relationship between OFH and mental health outcomes (15, 22, 23), as well as between LS and mental health (44). This could be accomplished by relying on robust research methods, including panel designs, estimation of fixed effects only based on within-person variation, and controlling for reverse causation (127, 128).

Data for this study were obtained from a convenience sample of individuals with psychiatric diagnoses seeking employment. Furthermore, people seeking employment are less likely to experience high levels of impairment, relative to people with psychiatric diagnoses not seeking employment. Hence, our findings are not necessarily generalizable to all people with psychiatric diagnoses. Nevertheless, given our high response rate and diverse sample (e.g., psychiatric diagnoses, racial/ethnic identities), our key findings may be applicable to individuals with psychiatric diagnoses in publicly funded supported employment programs.

This study utilized the Adult Hope Scale, which is considered a measure of trait or dispositional hope. It is theorized to be a relatively stable indicator associated with long-lasting personality traits (68). Research suggests that hope, experiences of goal attainment, and the environment are likely to interact, such that “trait” hope may increase in response to new experiences of success in achieving goals and environments perceived as conducive to this effect (129). Nevertheless, it is likely that the estimates in our study may be significantly different from those obtained from a “state” measure of hope (e.g., State Hope Scale), which may be more responsive to situational changes. By relying on the Adult Hope Scale, our study may have underestimated the relationships between hope, its components, OFH and SFH, and LS. Future research should examine the mediational paths explored in this study utilizing hope measures that may be more sensitive to change over time as well as those that tap into emotional dimensions of hope such as the Herth Hope Index (120).

Our finding about the weak and non-significant mediating role of hope pathways may have been biased by our study’s assumption that hope agency and pathways are contemporaneous and correlated, but do not have a causal effect on each other. If hope agency or hope pathways mediated the effect of the other on LS, when introduced as predictors in the same equation, the effect of the causal factor would be diminished or eliminated. The overall evidence in our study, however, suggests that the pathways component of hope is not likely to play a role as mediator, since the bivariate association between OFH and hope pathways is very weak and not statistically significant. Nevertheless, future research should explore the temporal and theoretical relationship between agency and pathways.

Regarding our estimates about the effects of covariates on OFH, it must be noted that diagnoses in our study were self-reported and relied on the person’s recall over their full lifetime, possibly leading to measurement error. It is also possible that diagnosis may be associated with unobserved characteristics predisposing to OFH. Thus, our findings on the effect diagnoses on OFH should be considered preliminary and in need of confirmation. Limited statistical variability in income and employment status in our relatively small sample, in addition to our broad economic measures, may have limited our ability to detect associations between income and employment with other psychological wellbeing outcomes. For instance, there was a small non-significant effect of employment status on OFH (*B* = –0.17 SD), which may have been detected with more statistical power. In addition, our income measure may not fully reflect the resources available to an individual. We could not obtain reliable data on household income for a substantial proportion of respondents. Hence, we relied only on individual income and not household income, which may be more predictive of OFH and its effects on psychological wellbeing (130).

## Conclusion

Taken together, our findings provide evidence about the strong effect of objective and SFH on the LS of unemployed and underemployed individuals with psychiatric diagnoses. Our findings especially underscore the role that the subjective experience of financial hardship, as well as the agency component of hope, play as mediators of the relationship between OFH and LS in this population.

Effective economic empowerment interventions to address financial hardship as a social determinant of mental health require integrated strategies to tackle objective and subjective dimensions alike. Interventions should include strategies that improve the subjective experience of financial hardship, for example, by fostering financial satisfaction and reducing financial shame. This study also highlights the importance of building financial agency, which may include strategies to enhance self-efficacy and optimism about one’s financial future. Peer-led interventions are especially well positioned to build financial agency, since research has consistently shown that hope is an evidence-based outcome of peer-led interventions. Interventions must also integrate strategies and approaches that address objective indicators of financial hardship, such as the inability to meet basic needs, housing unaffordability, and indebtedness; these include professional financial counseling to alleviate indebtedness and build credit and services to meet basic needs (e.g., housing, food). Supported employment programs, such as evidence-based Individual Placement Support programs (131), ought to consider integrating economic empowerment strategies in tandem with employment and clinical supports.

Future research should examine the relationships between OFH and SFH, hope, LS, and subjective wellbeing on larger samples using longitudinal designs that can better establish causal and dynamic effects. Future research should also develop and test the feasibility, acceptability, and efficacy of interventions that integrate supports to address objective and subjective dimensions of financial hardship.

## Data Availability Statement

The original contributions presented in this study are included in the article/supplementary material, further inquiries can be directed to the corresponding author.

## Ethics Statement

The studies involving human participants were reviewed and approved by the New York State Psychiatric Institute, Columbia Irving University Medical Center. Written informed consent for participation was not required for this study in accordance with the national legislation and the institutional requirements.

## Author Contributions

OJ-S: study design, data collection oversight, analysis plan, data analysis, and final draft. RP: literature review, analysis plan, data interpretation, framing discussion, tables, figures, and final draft editing. IM: data cleaning and initial data analysis, initial drafts. MW: senior oversight of data analysis and SEM expert advice, methods section, and final draft editing. HG: statistical advice, methods section, and final draft editing. PM-B: data analysis plan and data interpretation. PM-B, TL, MK, and LV: study and survey design, final draft editing. MS: study design and final draft editing. AC: literature review and final draft editing. EJ: hope theory, data interpretation, discussion and implications for practice, and final draft editing. TL and MK: supervised fieldwork, implications for practice, and final draft editing. LV: participant recruitment and interviews, final draft editing. RL-F: senior research oversight, data analysis plan, data interpretation, framing discussion, and final draft editing. All authors contributed to the article and approved the submitted version.

## Conflict of Interest

All authors declare that the research was conducted in the absence of any commercial or financial relationships that could be construed as a potential conflict of interest.

## Publisher’s Note

All claims expressed in this article are solely those of the authors and do not necessarily represent those of their affiliated organizations, or those of the publisher, the editors and the reviewers. Any product that may be evaluated in this article, or claim that may be made by its manufacturer, is not guaranteed or endorsed by the publisher.
